# Low-dose interleukin-2 in patients with mild to moderate Alzheimer’s disease: a randomized clinical trial

**DOI:** 10.1186/s13195-025-01791-x

**Published:** 2025-07-04

**Authors:** Alireza Faridar, Nazaret Gamez, Daling Li, Yanling Wang, Reena Boradia, Aaron D. Thome, Weihua Zhao, David R. Beers, Jason R. Thonhoff, Mohammad O. Nakawah, Gustavo C. Román, John J. Volpi, Jon B. Toledo, Michael George, Charles S. Davis, Belen Pascual, Michael Grundman, Joseph C. Masdeu, Stanley H. Appel

**Affiliations:** 1 Stanley H. Appel Department of Neurology, Houston Methodist Research Institute, Houston, TX 77030 USA; 2 Department of Pharmacy, Houston Methodist Hospital, Houston, TX 77030 USA; 3 CSD Biostatistics, Inc., 1005 W. Soft Wind Place, Tucson, AZ 85737 USA; 4 Global R&D Partners, LLC, 13236 Haxton Place, San Diego, CA 92130 USA; 5 Department of Neurosciences, University of California, San Diego, 92037 USA; 6 Stanley H. Appel Department of Neurology, Academic Institute, 6560 Fannin Street, Suite 750, Houston, TX 77030 USA; 7 Stanley H. Appel Department of Neurology, Johnson Center for Cellular Therapeutics, 6565 Fannin Street, Suite P3-201, Houston, TX 77030 USA

**Keywords:** Alzheimer’s Disease, Inflammation, Immunotherapy, Clinical trial, Treg, Immune system

## Abstract

**Background:**

We previously documented that regulatory T cells (Tregs) immunomodulatory mechanisms are compromised in Alzheimer’s disease (AD), shifting the immune system toward a pro-inflammatory response. However, Tregs are a potentially restorable therapeutic target in AD. In this study, we evaluated the safety and efficacy of two dosing frequencies of low-dose Interleukin-2 (IL-2) in expanding Tregs to modify disease progression in AD individuals.

**Methods:**

In this phase 2a, randomized, double-blind, placebo-controlled study, 38 participants were assigned to receive subcutaneous IL-2 (10^6 IU/day) for five days, administered either every 4 weeks (IL-2 q4wks) or every 2 weeks (IL-2 q2wks), versus placebo, for 21 weeks, followed by 9 weeks of observation. The primary endpoints were the incidence and severity of adverse events. For the secondary endpoints, changes in Treg numbers and suppressive functions were evaluated. Exploratory endpoints included changes in plasma inflammatory mediators, CSF AD-related biomarkers, and clinical scales.

**Results:**

Of the 38 participants, 9 received IL-2 q4wks, 10 received IL-2 q2wks, and 19 received placebo. All participants completed the trial with no serious adverse events or deaths. Both IL-2 dosing regimens increased Treg numbers and suppressive function, but IL-2 q4wks treatment exhibited superiority in enhancing Treg percentage and Foxp3 mean fluorescent intensity. In longitudinal analysis of 45 inflammatory mediators, IL-2 q4wks administration demonstrated greater efficacy in alleviating the plasma inflammatory mediators CCL2, CCL11, and IL-15, while enhancing IL-4 and CCL13 levels. A significant improvement in CSF Aβ42 levels (*p* = 0.045 vs. placebo) on Day 148 was observed following IL-2 q4wks administration, compared to placebo. While CSF NfL increased by 217 pg/ml in placebo recipients, it remained stable in the IL-2 q4wks group (*p* = 0.060, IL-2 q4wks vs. placebo). The adjusted mean change from baseline in the ADAS-cog score at week 22 indicated a trend toward slower clinical progression in IL-2 q4wks recipients compared to placebo (*p* = 0.061).

**Conclusions:**

The IL-2 immunotherapeutic strategy was safe and well-tolerated. IL-2 q4wks effectively expanded Treg populations, leading to modification in inflammatory mediators and CSF Aβ42 levels, while also showing promising trends on clinical scales. These findings provide a foundation for further investigation of low-dose IL-2 as a potential treatment for Alzheimer’s Disease.

**Trial registration:**

ClinicalTrials.gov Identifier: NCT06096090, Registration Date: 10-17-2023.

**Supplementary Information:**

The online version contains supplementary material available at 10.1186/s13195-025-01791-x.

## Background

The discovery of risk genes involved in inflammation signaling suggests that inflammation plays a critical role in the onset and progression of Alzheimer’s disease (AD) and could serve as a new target for therapeutic intervention [1, 2]. Preclinical findings indicate that glial cells are initial responders to neuronal release of pathological forms of amyloid-β and tau proteins [3–7]. Persistent glial activation promotes the release of inflammatory mediators leading to a self-propagating cascade of synaptic dysfunction, neuronal injury, and cell death [6, 8, 9]. In the presence of AD pathology, the structural integrity of the blood–brain barrier is impaired [10, 11], allowing substantial crosstalk between central and peripheral immune systems and enabling peripheral immune cells to contribute to disease progression [12–14].

Regulatory T cells (Tregs) constitute a subset of T cells that play a protective role by modulating blood and brain inflammation [15, 16]. We previously documented that Treg immunomodulatory mechanisms are compromised in AD individuals [17], associated with activation of systemic myeloid populations, expansion of cytotoxic T cells and upregulation of inflammatory mediators [13, 17, 18]. Overexpression of inflammatory mediators such as CCL2, CCL4, CCL11, and CXCL6 in AD further facilitates the infiltration and migration of systemic inflammatory immune populations toward the central nervous system (CNS) and exacerbates neuroinflammation [19–22].

Accumulating preclinical and clinical evidence suggests that Tregs may be a modifiable therapeutic target. Interleukin 2 (IL-2), originally described as the main T-cell growth factor, has been used in standard high doses to activate cytotoxic T cells and natural killer cells in cancer therapy [23]. However, at low doses, IL-2 selectively binds to Treg populations due to the constitutive expression of the high-affinity IL-2 receptor complex on the Treg membrane [24]. Low-dose IL-2 administration has been studied in various indications and demonstrated a favorable safety profile across multiple clinical contexts [25–27]. In a prior study we conducted, low-dose IL-2 treatment enhanced AD Treg suppressive function in vitro [17]. In preclinical mouse models, expansion of peripheral Tregs effectively suppressed neuroinflammation and alleviated AD pathology [14, 28]. To translate this treatment strategy to the clinical setting, subcutaneous low-dose IL-2 treatment protocol was developed and administered to AD individuals in a phase 1 feasibility study [29]. Five-day courses of subcutaneous IL-2, administered every 4 weeks safely expanded Treg populations, suppressed monocytes inflammatory markers, and reduced plasma inflammatory cytokines and chemokines in the enrolled AD participants [29]. To confirm and expand these findings, we conducted a phase 2a double-blind, randomized clinical trial to assess the safety and potential efficacy of low-dose IL-2 treatment strategies in participants with mild to moderate AD. In this proof-of-concept trial, we assessed the impact of two different dosing frequencies of 5-day cycles of IL-2 treatment (every 4 weeks and every 2 weeks) on peripheral Treg populations, plasma inflammatory mediators, CSF AD-related biomarkers, and cognitive and functional endpoints.

## Methods

### Study design and participants

This study was a phase 2a, proof-of-concept, randomized, double-blinded, three-arm, placebo-controlled trial to evaluate two different dosing frequencies of low-dose IL-2 treatment in AD subjects. The trial was conducted according to the Declaration of Helsinki following ethics approval from the Institutional Review Board at the Houston Methodist Research Institute. The Nantz National Alzheimer Center (NNAC) was the sole recruitment site for this trial. All patients signed informed consent prior to study enrolment. Enrollment began in January 2022 and ended in October 2023, with database lock and unblinding occurring in June and July 2024, respectively. Two main inclusion criteria were age between 50 and 86 years and, a Mini-Mental State Examination (MMSE) score of 12–26. All participants had lumbar puncture at the screening visit to confirm an AD diagnosis based on cerebrospinal fluid (CSF) biomarkers, defined as an A-beta 42 (Aβ42) to total tau index (ATI) of < 0.8 and a p-tau181 level > 68 pg/mL (ADmark® assay, Athena Diagnostic) [30, 31]. Lumbar puncture was repeated following completion of the treatment phase on day 148 to monitor the changes in CSF biomarkers. An independent medical, data and safety officer provided trial oversight.

### Randomization and intervention

**Fig. 1:**
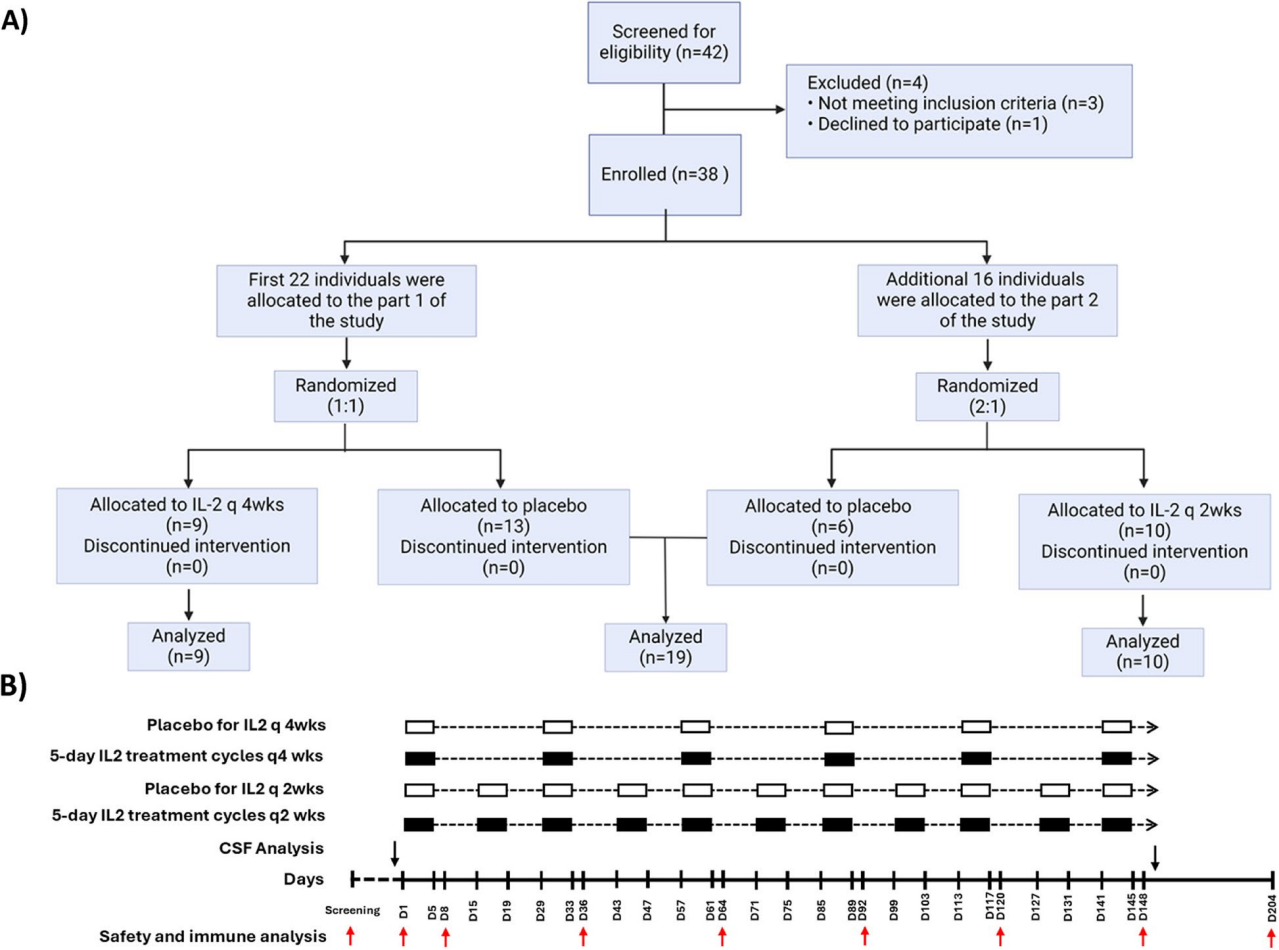
Flow diagram and intervention. **A** Subjects screened for the study and those eligible were enrolled. The first 22 participants were randomized to receive five-day courses of subcutaneous Interleukine-2 (IL-2) every 4 weeks (IL-2 q4wks), or placebo (1:1 ratio). 16 additional participants were randomized to receive IL-2 every two weeks (IL-2 q2wks) or placebo (2:1 ratio). All enrolled individuals completed the treatment phase and underwent assessment for the primary safety endpoints. **B** Schematic representation of the 5-day IL-2 administration cycles (black rectangles) in the two treatment arms-IL-2 q4wks and IL-2 q2wks-as well as the corresponding placebo administrations (white rectangles). Blood samples (red arrows) for safety and immune analysis are drawn on day 1 (before IL-2 administration), days 8, 36, 64, 92, 120, and 148 during the treatment phase, as well as on day 204 during the post-treatment phase

A total of 38 participants were enrolled in the study. The first 22 participants were randomized (1:1 ratio intended) to five-day courses of subcutaneous low-dose IL-2 (10^6^ UI/day) treatment or placebo administered every 4 weeks for a total of 21 weeks. In the next step, an additional 16 participants were randomized (2:1 ratio intended) to five-day-courses of subcutaneous IL-2 (10^6^ UI/day) or placebo administered every two weeks for a total of 21 weeks (Fig. 1A-B). Subjects were followed for 9 weeks after the last five-day course of treatment for a total of 30 weeks. To ensure the integrity of the study and minimize bias, a rigorous blinding and randomization process was implemented. Randomization sequences were generated using a computer-based algorithm to allocate participants to study treatment or placebo, while maintaining stratification factors [sex (male, female) and baseline MMSE score (12–18 and 19–26)] to ensure balanced group assignment. Study participants, investigators, and outcome assessors remained masked to treatment allocation throughout the trial. Recombinant human IL-2 (Proleukin) was purchased from Clinigen and was dispensed by the Houston Methodist Investigational Pharmacy. Under aseptic conditions in a laminar flow hood, vials of Proleukin were reconstituted with 1.2 mL of sterile water and further diluted with 5% Dextrose Water (D_5_W) for injection. The prepared solution was stored in 1 ml Becton–Dickinson plastic syringes at 2° to 8 °C, with each 1 mL syringe containing one dose at 0.31 mL, equivalent to 10^6^ UI. The syringes were maintained under refrigeration until use. The placebo consisted of D_5_W and the appearance and color of the placebo vials were similar to those of the active drug.

### Outcomes

The primary objective of this study was to assess the safety and tolerability of low-dose IL-2 administration in AD participants. Safety measures were monitored throughout the trial by characterizing adverse events (AEs), chemistry and hematology laboratory values, vital signs, and physical and neurological examinations. AEs were classified by severity as mild (requiring minimal or no treatment and not interfering with daily activities), moderate (causing a low level of inconvenience or concern and potentially interfering with functioning), or severe (interrupting usual daily activities and potentially requiring systemic therapy or medical intervention). Serious adverse events (SAEs) were also defined as events resulting in death, life-threatening experiences, hospitalization or prolongation of hospitalization, or persistent or significant disability/incapacity. Tolerability was defined as the percentage of participants who completed the treatment period of the trial.

The secondary objective of this study was to investigate the impact of two different dosing frequencies of IL-2 treatment on the peripheral Treg population in AD individuals. Blood samples were obtained serially from enrolled individuals on day 1 (before IL-2 administration), day 8 (3 days following the first cycle of IL-2 administration) and then every four weeks on days 36, 64, 92, 120 and 148 (3 days following the latest cycle of IL-2 administration) throughout the treatment phase as well as 9 weeks after completion of the treatment on day 204 (Fig. 1B). The percentage and immunophenotype of peripheral immune populations were assessed by flow cytometry, as previously described [17, 29]. We applied a gating strategy that allows the identification of Tregs by an established phenotype of CD4^+^FoxP3^+^CD25^high^ (eFigure-1). The percentages of Tregs and other immune cell populations were measured at each timepoint, and changes from baseline were calculated as the difference: (% after treatment) – (% at baseline) for each time point to assess treatment-related effects over time. To evaluate changes in Treg immunosuppressive function, Tregs and responders T cells (Tresps) were isolated using the CD4^+^CD25^+^ Regulatory T Cell Isolation Kit (Miltenyi Biotec) according to the manufacturer’s instructions. Tregs and Tresps were co-cultured at a 1:1 ratio, and Treg-mediated suppression of Tresp proliferation was assessed as previously described [17].

The exploratory objective of this study was to investigate the impact of IL-2 administration on plasma inflammatory mediators, CSF AD-related biomarkers as well as clinical scales in AD participants. Plasma inflammatory mediators including chemokine and cytokine levels were analyzed longitudinally with the Olink® Target 48 Cytokine panel (Olink Proteomics, Uppsala, Sweden). This panel uses a multiplex proximity extension assay (PEA) technology to enable measurement of 45 selected cytokines and chemokines simultaneously from small volumes of plasma, through a service provided by the manufacturer. AD CSF biomarkers [Aβ42, phosphorylated tau 181 (p-tau181), glial fibrillary acidic protein (GFAP), and neurofilament light chain (NfL)], were measured at baseline and day 148, using the Single Molecule Array (Simoa) technology on the Quanterix HD-X platform. To determine possible changes in the clinical status, cognitive scales including Alzheimer Disease Assessment Scale–Cognitive subscale (ADAS-Cog), Clinical Dementia Rating Scale Sum of Boxes (CDR-SB) and Alzheimer’s Disease Cooperative Study-Clinical Global Impression of Change (ADCS-CGIC) were also assayed at the screening visit, day 64 and day 148.

### Statistical analysis

All statistical tests were conducted using two-sided tests at the alpha = 0.05 level of significance, with no adjustments for multiplicity. Apart from demographic and AE comparisons, all variables were analyzed as the change from screening/baseline with a covariate for the corresponding screening/baseline value.

#### Demographic variables

One-way analysis of variance (ANOVA) models with treatment group (3 levels) as a factor were used to compare the baseline distributions of quantitative variables. In addition, one way ANOVA models were used to compare each of the two active arms to its corresponding placebo arm. Fisher’s exact tests were used to compare the distributions of categorical variables. If the overall comparison was statistically significant pairwise between-group comparisons were made using Fisher’s exact tests. Fisher’s exact tests were also used to compare each of the two active arms to its corresponding placebo arm.

#### Safety

The incidence and severity of adverse events, changes in clinical laboratory testing (serum chemistry, hematology), and changes in vital signs and body weight were summarized descriptively. For each type of adverse event, Fisher’s exact tests were used to test the null hypothesis that the rates in the three groups were equal. If the overall test was statistically significant (*p* < 0.05), pairwise between-group comparisons were made using Fisher’s exact test. The distributions of each laboratory variable were compared at screening using one-way ANOVA models with treatment group (3 levels) as a factor. The distributions of the change from screening to last visit were compared using analysis of covariance (ANCOVA) models with treatment group (3 levels) as a factor and the screening value of the corresponding variable as a covariate.

#### Efficacy

For efficacy endpoints including immune analysis and CSF AD biomarkers, analysis of covariance (ANCOVA) models with the change from baseline to the specified time point as the dependent variable, treatment group (3 levels) as a factor, and the baseline value of the corresponding endpoint as a covariate were used. Comparisons between each active arm separately versus placebo were tested using these models. For Treg percentage, FOXP3 mean fluorescence intensity (MFI), CD25 MFI and Treg suppression values, post hoc comparisons were made between the two active arms. Additional exploratory analyses were conducted using mixed mode for repeated measures (MMRM) for clinical assessment endpoints collected at more than one post-baseline visit. In these models, the change from baseline was analyzed using a MMRM model with treatment, visit and treatment-by-visit interaction as factors. The baseline assessment scores were included as a covariate. All post-baseline analysis visits, performed during the double-blind period, were included in the model. An unstructured covariance matrix was used to model the within-participant errors. The Kenward-Roger approximation was used to estimate degrees of freedom. In comparing between arms, least Square (LS) mean differences in change from baseline were presented along with associated standard error of the mean (SEM). The CGIC variables were analyzed using the Cochran-Mantel–Haenszel mean score test. The analysis of clinical endpoints was evaluated independently and prior to any access to immune biomarker data to ensure that the interpretation of clinical findings was not influenced by biomarker results.

## Results

### Study population and primary endpoints

Participants were recruited from the memory clinic at NNAC and had previously undergone a thorough workup before receiving an AD diagnosis. Forty-two participants were screened. Thirty-eight AD participants (mean age: 70.5 years; 23 women [60.5%], baseline MMSE 18.0) were enrolled in this three-arm study and included in the intent-to-treat analysis. The baseline demographic characteristics of enrolled participants are presented in Table 1. Nine participants were assigned to receive IL-2 q4wks, 10 subjects received IL-2 q2wks, and 19 subjects received placebo injections (Fig. 1A). Of these 19 placebo subjects, 13 participants corresponded to Part 1 of the study and received placebo every 4 weeks, while 6 participants received placebo every 2 weeks (supplementary etable-1). Baseline characteristics including sex distribution, race, educational levels, concomitant acetylcholinesterase/memantine treatment and antidepressant intake, APOE status, or cognitive scale scores were comparable across the three study arms, with no significant differences. The only observed difference was the mean age of participants, which was higher in the IL-2 q2wks arm, compared to the IL-2 q4wks (*p* = 0.036) and placebo arms (*p* = 0.015). All enrolled subjects completed the 21-week treatment phase. The primary study endpoints were the incidence and severity of adverse events. In this study, there were no serious adverse events. The overall incidence of AEs was comparable between groups (IL-2 q4wks: 66%, IL-2 q2wks: 80%, Placebo: 73%). Adverse events with an incidence > 10% in any of the treatment groups included elevated eosinophil counts, erythema at the injection site, decreased hemoglobin levels, gastrointestinal symptoms, falls, gout relapse and shingles (Table 2). An increased eosinophil count and erythema at the injection site occurred at significantly higher rates in the treatment arms compared to placebo. The means of safety laboratory values at baseline and at the end of the treatment phase were also analyzed. Final mean values for all variables remained within normal laboratory ranges, except for eosinophil percentage (%), which increased in the IL2 q2wk arm, compared to placebo (IL-2 q4wks: baseline: 1.6 ± 0.8%, week 22: 3.5 ± 1.8%; IL-2 q2wks: baseline: 3.4 ± 1.7%, week 22: 10.4 ± 7.9%; Placebo: baseline: 2.2 ± 2.2%, week 22: 2.4 ± 2.0%; *p*-value IL-2 q4wks vs. placebo: 0.443, *p*-value IL-2 q2wks vs. placebo < 0.001) (eTable-2). No major changes in vital signs and weight were noted across the three arms throughout the 6-month treatment period.

**Table 1:** Baseline demographic characteristics of the clinical trial participants

Characteristics of the participants at baseline					
Characteristics	All Subjects (*n* = 38)	IL-2 q 4wks (*n* = 9)	IL-2 q 2wks (*n* = 10)	Placebo (*n* = 19)	*p*-Value
Age-yr (MEAN ± SD)	70.5 ± 7.7	68.7 ± 6.9	75.9 ± 6.5^a^	68.6 ± 7.6	0.035
Early vs. Late Onset- no. (%)					
Early Onset	12 (31.5%)	4 (44.4%)	1 (10%)	7 (36.8%)	
Late Onset	26 (68.4%)	5 (55.5%)	9 (90%)	12 (63.1%)	
Sex- no. (%)					1.000
Female	23 (60.5%)	6 (66.6%)	6 (60.0%)	11 (58.8%)	
Male	15 (39.4%)	3 (33.3%)	4 (40.0%)	8 (42.1%)	
Race/Ethnicity-no. (%)					0.895
White/non-Hispanic	34 (89.4%)	9 (100%)	9 (90.0%)	16 (84.2%)	
White/Hispanic	3 (7.8%)	0 (0%)	1 (10.0%)	2 (10.5%)	
Black	1 (2.6%)	0 (0%)	0 (0.0%)	1 (5.2%)	
Education- no. (%)					0.840
High school	4 (10.5%)	0 (0%)	2 (20.0%)	2 (10.5%)	
College	24 (63.1)	6 (66.6%)	6 (60.0%)	12 (63.1%)	
Post-grad	10 (26.3)	3 (33.3%)	2 (20.0%)	5 (26.3%)	
Clinical Subtypes					1.000
Amnestic	31 (81.5%)	7 (77.7%)	8 (80%)	16 (84.2%)	
Non-Amnestic	7 (18.4%)	2 (22.2%)	2 (20%)	3 (15.7%)	
Concomitant medications					1.000
Current Cognitive Tx (AChEI/Memantine)	31 (81.5%)	6 (66.6%)	9 (90%)	16 (84.2%)	
Antidepressants	22(56%)	5(55.5%)	6(60%)	11 (57.8%)	
APOE ε4 status- no. (%)					0.508
Noncarrier	11 (28.9%)	3 (33.3%)	2 (20.0%)	6 (31.5%)	
Carrier-Heterozygotes	15 (39.4%)	4 (44.4%)	6 (60.0%)	5 (26.3%)	
Carrier-Homozygotes	12 (31.5%)	2 (22.2%)	2 (20.0%)	8 (42.1%)	
Cognitive scales (MEAN ± SD)					
MMSE score	18.0 ± 3.1	18.7 ± 2.9	18.5 ± 2.8	17.3 ± 3.4	0.540
CDR-SOB score	4.6 ± 2.8	4.7 ± 3.1	4.3 ± 2.3	4.8 ± 3.0	0.892
ADAS-Cog score	33.5 ± 13.2	31.2 ± 12.8	30.7 ± 8.5	36.0 ± 15.3	0.511
CDR-Global distribution					0.931
CDR 0.5	20 (52.6%)	5(55.5%)	5 (50%)	10 (52.6%)	
CDR 1	16 (42.1%)	4 (44.4%)	5 (50%)	7 (36.8)	
CDR 2	2 (5.2%)	0 (0%)	0 (0%)	2 (10.5%)	

^a^IL2 q2wks vs. Placebo *P* = 0.015 and IL2 q2wks vs. IL2 q4wks *p* = 0.036

**Table 2:** Overall summary of adverse events

Adverse Events Summary				
Events	IL-2 q4wks (*n* = 9)	IL-2 q2wks (*n* = 10)	Placebo (*n* = 19)	*p*-Value
Subjects with at least one AE:	6(66%)	8(80%)	14(73%)	
Death	0	0	0	-
Serious AEs	0	0	0	-
Non-serious Moderate AEs				
Fall	1(11%)	-	1 (5%)	0.730
Gallstone	1(11%)	-	-	0.237
Covid Pneumonia	-	-	1 (5%)	1.000
Musculoskeletal pain	-	-	1 (5%)	1.000
Gout relapse	1(11%)	-	-	0.237
Presyncope	-	-	1 (5%)	1.000
Non-serious Mild AEs				
Increased eosinophil count	2(22%)	3 (30%)	-	**0.036**
Erythema at injection site	1(11%)	3 (30%)	-	**0.031**
Decreased hemoglobin level	1(11%)	1 (10%)	5 (26%)	0.641
Nausea/Vomiting/Abdominal pain	-	2 (20%)	3 (15.7%)	0.566
Leukopenia	-	1 (10%)	3 (15.7%)	0.792
Fall	1(11%)	-	1 (5%)	0.730
Covid pneumonia	-	-	2 (10.5%)	0.730
Hypokalemia	-	1 (10%)	1 (5%)	1.000
Presyncope	-	-	1 (5%)	1.000
Elevated TSH	-	-	1 (5%)	1.000
Shingles	1(11%)	-	-	0.237
Cough	-	-	1 (5%)	1.000
Leg sciatica	-	1	1 (5%)	1.000
Fatigue	-	-	1 (5%)	1.000
Chest pain	-	1 (10%)	-	0.500
Elevated troponin	-	1 (10%)	-	0.500

### Secondary outcomes

**Fig. 2:**
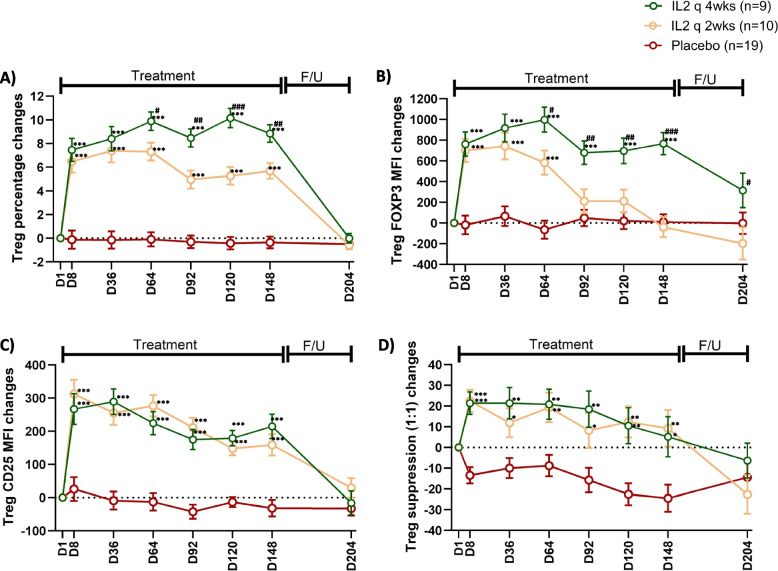
Effect of low-dose IL-2 treatment on Treg population. **A** The percentage of CD4^+^ T cells that were FOXP3^+^CD25 ^high^ (i.e., Tregs) was assessed by flow cytometry at baseline (D1), throughout the treatment phase on D8, D36, D64, D92, D120 and D148, and at the end of the follow-up (F/U) phase at D204 among three arms: IL-2 every 4 weeks (IL-2 q4wks), IL-2 every 2 weeks (IL-2 q2wks), and placebo. **B** Foxp3 mean fluorescence intensity (MFI) and **C** CD25 MFI in the Treg population were also monitored. **D** The suppressive function of isolated Tregs on corresponding T responder (Tresp) cell proliferation in vitro, at a 1:1 Treg to Tresp ratio, was assessed. D = Day. Data represent mean changes from baseline ± SE. Comparison across the three arms were compared using ANCOVA. *P*-values are represented as *p < 0.05, **p < 0.01, and ***p < 0.001 for IL-2 q4wks or IL-2 q2wks versus placebo, and #*p* < 0.05, ##*p* < 0.01, and ###*p* < 0.001 for IL-2 q4wks versus IL-2 q2wks

The increases in the percentage of CD4^+^FoxP3^+^CD25^high^ Treg from the baseline levels were highly significant (*p* value < 0.001) across all the six timepoints (D8, D36, D64, D92, D120, D148) throughout the treatment phase in both the IL-2 q2wk and IL-2 q4wk arms, compared to the placebo arm. Of interest, on days 64, 92, 120, and 148, the changes from baseline in Treg percentage were greater in the IL-2 q4wks arm than IL-2 q2wks (IL-2 q4wks vs. IL-2 q2wks; D8: *P* = 0.469, D36 *P* = 0.474, D64: *P* = 0.025, D92: *P* = 0.003, D120: *P* < 0.001, D148: *P* = 0.003) (Fig. 2A). In further analysis of Treg population, Foxp3 MFI significantly increased from baseline following IL-2 administration until day 64 in both treatment arms, compared to placebo. However, while FoxP3 MFI remained elevated through the end of the treatment in the IL-2 q4wks arm, levels in the IL-2 q2wks arm decreased toward placebo levels by days 92, 120 and 148 (IL-2 q4wks vs. Placebo; D8: *P* < 0.001, D36: *P* < 0.001, D64: *P* < 0.001, D92: *P* < 0.001, D120: *P* < 0.001, D148: *P* < 0.001; IL-2 q2wks vs. Placebo; D8: *P* < 0.001, D36: *P* < 0.001, D64: *P* < 0.001, D92: *P* = 0.078, D120: *P* = 0.069, D148: *P* = 0.694) (Fig. 2B). These findings suggest a reduced efficacy of the IL-2 q2wks regimen in expanding Treg population and increasing their FOXP3 MFI during the latter part of the treatment phase. Participants in the IL-2 q2wks arm were, on average, 7.2 years older than those in the IL-2 q4wks arm, suggesting that older age might contribute to reduced Treg proliferative capacity in the IL-2 q2wks group. However, no significant correlations were observed between age and changes in Treg percentage (Spearman *r* = –0.15, *p* = 0.36) or suppressive function (Spearman *r* = 0.11, *p* = 0.60) from baseline to week 22 following IL-2 administration (*n* = 19; data not shown). Treg CD25 MFI (Fig. 2C) and Treg suppression of Tresp proliferation (Fig. 2D) also increased from baseline following both IL-2 q2wks and IL-2 q4wks administration. In contrast to Treg percentage and FOXP3 MFI, no major differences in changes from baseline in CD25 MFI and Treg suppression were observed when comparing the two treatment arms. In the post-hoc analysis of Treg-related variables (Treg percentage, FOXP3 and CD25 MFI, and Treg suppression), similar results were observed whether each treatment arm was compared with its corresponding placebo group or with the total placebo group (eTable-3). The percentages of effector T cells including CD4^+^CD25^low^ Tresps (eFigure 2-A) and CD8^+^ T cells (eFigure 2-B) populations did not differ significantly from baseline following IL-2 q2wks or IL-2 q4wks administrations. In evaluating CD3^−^CD56^+^ natural killer cells (NKCs), a significant increase was observed on day 8 in the IL-2 q2wks arm and on day 120 in the IL-2 q4wks arm, compared to placebo (eFigure 2-C).

### Exploratory outcomes

**Fig. 3:**
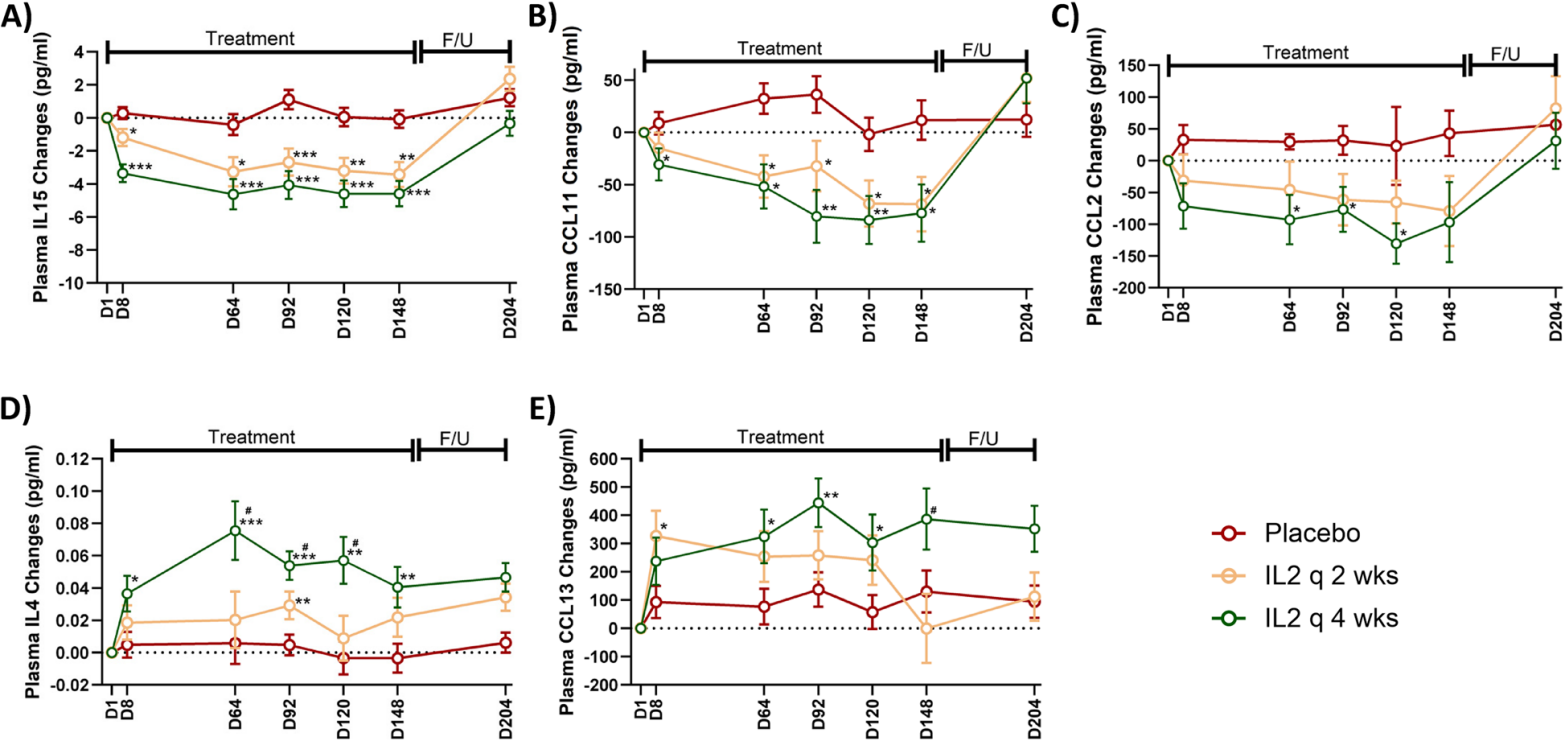
Effect of low-dose IL-2 treatment on plasma inflammatory biomarkers. Plasma levels of immune-related biomarkers, including IL15 (**A**), CCL11 (**B**), CCL2 (**C**), IL-4 (**D**) and CCL13 (**E**) were measured longitudinally using Olink protein analysis at baseline (D1), throughout the treatment phase (D8, D64, D92, D120, and D148), and at the end of the follow-up (F/U) phase on D204 across three study arms: IL-2 every 4 weeks (IL-2 q4wks), IL-2 every 2 weeks (IL-2 q2wks), and placebo. D = Day. Data represent mean changes from baseline ± SE. Comparison across the three arms were compared using ANCOVA. *P*-values are represented as *p < 0.05, **p < 0.01, and ***p < 0.001 for IL-2 q4wks or IL-2 q2wks versus placebo, and #*p* < 0.05 for IL-2 q4wks versus IL-2 q2wks

Exploratory endpoints included changes from baseline in plasma inflammatory mediators, CSF AD-related biomarkers, and clinical scales following 21 weeks of IL-2 treatments. Using Olink analysis, 45 plasma inflammatory cytokines and chemokines were measured longitudinally at baseline, at five time points during the treatment phase (D8, D64, D92, D120, and D148), and 9 weeks after treatment completion on D204. The plasma levels of nine immune markers (IL1b, IL7, IL-2, IL13, IL-17A, IL-17F, IL33, TSLP and CSF2) were less than lowest quantifiable levels and excluded from the analysis. Both IL-2 q4wks and IL-2 q2wks administrations significantly suppressed plasma levels of the inflammatory cytokine IL-15 compared to placebo throughout the treatment phase (IL-2 q4wks vs. placebo: D8, *P* = 0.001; D64, *P* < 0.001; D92, *P* < 0.001; D120, *P* < 0.001; D148, *P* < 0.001; IL-2 q2wks vs. placebo: D8, *P* = 0.026; D64, *P* = 0.014; D92, *P* < 0.001; D120, *P* = 0.002; D148, *P* = 0.001) (Fig. 3A). Similarly, both IL-2 regimens reduced the plasma levels of chemokine CCL11 throughout the treatment phase (IL-2 q4wks vs. placebo: D8, *P* = 0.042; D64, *P* = 0.002; D92, *P* < 0.001; D120, *P* = 0.006; D148, *P* = 0.011; IL-2 q2wks vs. placebo: D8, *P* = 0.198; D64, *P* < 0.001; D92, *P* = 0.028; D120, *P* = 0.020; D148, *P* = 0.018) (Fig. 3B). IL-2 q4wks administration also significantly suppressed plasma levels of the macrophage/microglial activation chemokine CCL2, compared to placebo (IL-2 q4wks vs. placebo: D8, *P* = 0.022; D64, *P* = 0.034; D92, *P* = 0.017; D120, *P* = 0.040; D148, *P* = 0.079). However, this suppression was not statistically significant in the IL-2 q2wks arm (IL-2 q2wks vs. placebo: D8, *P* = 0.200; D64, *P* = 0.280; D92, *P* = 0.064; D120, *P* = 0.924; D148, *P* = 0.076) (Fig. 3C). Additionally, plasma levels of the anti-inflammatory cytokine IL-4 were significantly elevated in the IL-2 q4wks arm compared to placebo at all five time points during the treatment phase (IL-2 q4wks vs. placebo: D8, *P* = 0.030; D64, *P* = 0.004; D92, *P* < 0.001; D120, *P* = 0.002; D148, *P* = 0.009). IL-4 levels in the IL-2 q4wks arm were also significantly higher than those in the IL-2 q2wks arm at D64, D92, and D120 (IL-2 q4wks vs. IL-2 q2wks: D8, *P* = 0.238; D64, *P* = 0.030; D92, *P* = 0.047; D120, *P* = 0.018; D148, *P* = 0.277) (Fig. 3D). We also identified increased levels of plasma CCL13 chemokine at Day 8 in the IL-2 q2wks arm (*P* = 0.0331), and at Days 64 (*P* = 0.037), 92 (*P* = 0.007) and 120 (*P* = 0.042) in the IL-2 q4wks arm, compared to placebo. Additionally, plasma CCL13 levels at Day 148 were significantly higher in the IL-2 q4wks arm compared to the IL-2 q2wks arm (*P* = 0.024). No statistically significant longitudinal changes were observed in 31 other measured plasma immune markers (eFigure 3).

**Fig. 4:**
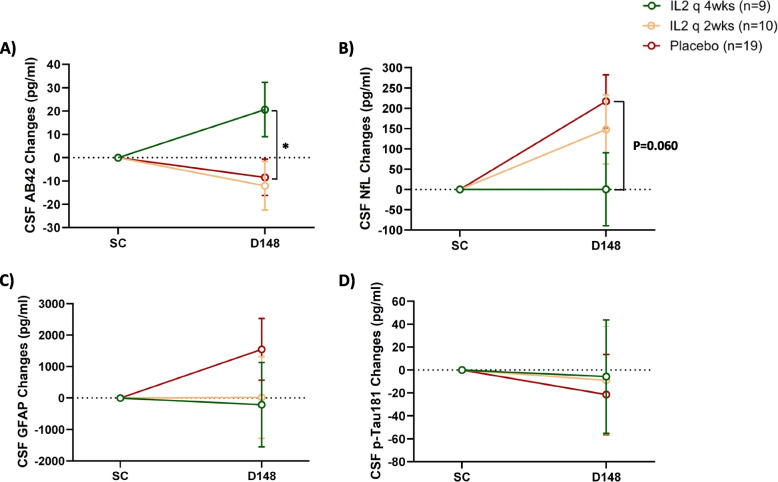
Effect of low-dose IL-2 treatment on Alzheimer’s disease biomarkers. Alzheimer’s disease (AD) cerebrospinal fluid (CSF) biomarkers, including A-beta 42 (Aβ42) (**A**), neurofilament light chain (NfL) (**B**), glial fibrillary acidic protein (GFAP) (**C**), and phosphorylated tau 181 (p-tau181) (**D**), were measured using the Simoa Quanterix assay at screening (SC) and at the end of the treatment phase on Day 148 (D148). Data represents mean changes from baseline ± SE. Comparisons across the three arms were made using ANCOVA. *P*-values are represented as *p < 0.05

AD CSF biomarkers were also measured at baseline and following completion of the treatment on day 148. IL-2 q4wks administration significantly elevated CSF Aβ42 levels by day 148, compared to the placebo group (*p* = 0.045), suggesting a normalizing effect on CSF Aβ42 levels following 21 weeks of IL-2 q4wks treatment. In contrast, changes in CSF Aβ42 levels in the IL-2 q2wks arm were comparable to those in the placebo group (Fig. 4A). While CSF NfL levels remained stable following IL-2 q4wks administration (change from baseline = 0.48 ± 89.92 pg/mL), they increased by 148.07 ± 85.21 pg/mL in the IL-2 q2wks arm and 217.38 ± 65.39 pg/mL in the placebo arm. In the comparison between the treatment arms and placebo, a trend toward stabilization of NfL levels was observed only with IL-2 q4wks treatment (IL-2 q4wks vs. placebo, *p* = 0.060; IL-2 q2wks vs. placebo, *p* = 0.523) (Fig. 4B). CSF GFAP levels, a biomarker of glial activation, slightly decreased following IL-2 q4wks administration (change from baseline: −214.10 ± 1339.91 pg/mL), remained almost stable in the IL-2 q2wks group (change from baseline: 17.41 ± 1297.49 pg/mL), but increased by 1548.99 ± 982.68 pg/mL in the placebo group. However, the changes in CSF GFAP in both the IL-2 q4wks and IL-2 q2wks groups were not statistically significant compared to the placebo arm (IL-2 q4wks vs. placebo, *p* = 0.295; IL-2 q2wks vs. placebo, *p* = 0.359) (Fig. 4C). No significant changes on CSF p-Tau181 levels were found after 21 weeks of IL-2 treatment (Fig. 4D).

**Fig. 5:**
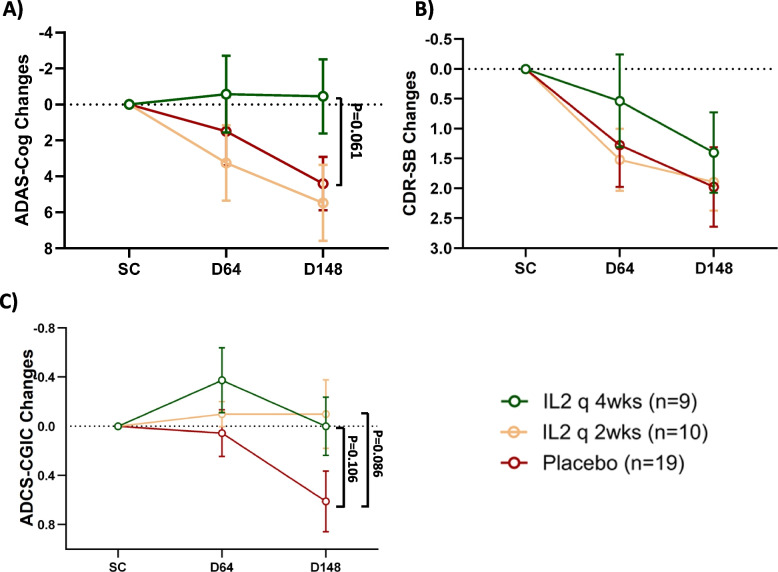
Effect of low-dose IL-2 treatment on clinical scales. Cognitive tests, including the Alzheimer’s Disease Assessment Scale–Cognitive Subscale (ADAS-Cog) (**A**), Clinical Dementia Rating Scale Sum of Boxes (CDR-SB) (**B**), and Alzheimer’s Disease Cooperative Study-Clinical Global Impression of Change (ADCS-CGIC) (**C**), were conducted at screening (SC), day 64 (D64), and day 148 (D148). Data represents mean changes from baseline ± SE. An increase in the scores in all these tests indicates cognitive deterioration. For ADAS-Cog and CDR-SB, comparisons across the three arms were made using the MMRM model. The CGIC variables were analyzed using the Cochran-Mantel–Haenszel mean score test

Clinical scales, including ADAS-Cog, CDR-SB, and ADCS-CGIC, were assessed at baseline, day 64, and day 148. ADAS-Cog scores on day 148 showed a slight improvement following IL-2 q4wks administration (change from baseline: −0.45 ± 2.06), while scores worsened in both the IL-2 q2wks (change from baseline: 5.16 ± 2.10) and the placebo (change from baseline: 4.48 ± 1.48) groups. A trend was detected toward slowing of clinical progression in the IL-2 q4wks arm compared to the placebo group (*p* = 0.061) (Fig. 5A). The change from baseline in CDR-SB on Day 148 was 1.40 ± 0.67 in the IL-2 q4wks group, 1.97 ± 0.66 in the IL-2 q2wks group, and 1.89 ± 0.48 in the placebo group, suggesting possible slower decline in CDR-SB scores following IL-2 q4wks treatment compared to the placebo group, however, this difference was not statistically significant (*p* = 0.548) (Fig. 5B). A trend toward reduced worsening of ADCS-CGIC scores was observed in both the IL-2 q4wks and IL-2 q2wks groups on day 148 compared to the placebo arm (IL-2 q4wks vs. placebo, *p* = 0.106; IL-2 q2wks vs. placebo, *p* = 0.086) (Fig. 5C). In a further analysis of clinical endpoints, APOE status was included as a factor in the models. The *p*-values were comparable to those from the analyses that did not include APOE status (Data not shown). Finally, to evaluate the treatment effect over a longer period following treatment completion, CDR-SOB and ADAS-Cog assessments were conducted in the post-treatment follow-up phase, on Day 204. Cognitive performances remained more favorable in the IL-2 q4wks arm compared to placebo. However, while ADAS-Cog scores showed some decline after the treatment phase, CDR-SOB scores further improved by 0.383 points (efigure-4).

## Discussion

This Phase 2a randomized, double-blind, placebo-controlled, three-arm study investigating two different dosing frequencies of low-dose IL-2 met the primary safety and tolerability endpoints. To our knowledge, this is the first report of a novel low-dose IL-2 treatment strategy in individuals with AD that has advanced to a Phase 2 trial. The subjects in the trial were well balanced with respect to most baseline characteristics. However, we noted that participants in the IL-2 q2wk group were on average about 7 years older than those in the other treatment groups. Although participants were randomly assigned to treatment in the trial, given the small sample size of the study, some imbalances in the participant baseline characteristics were bound to occur by chance. However, it cannot be completely ruled out that the treatment effects on systemic and central immune responses may have been influenced by differences in patients’ ages. No serious AEs or treatment-related deaths recorded across any study arm. Among non-serious mild AEs, increased eosinophil count and injection site erythema were more common in both IL-2 treatment groups compared to placebo, aligning with previous reports of known side effect of low-dose IL-2 immunotherapies in other clinical contexts [29, 32–34].

Consistent with findings from our earlier Phase 1 feasibility study [29], administering 5-day cycles of low-dose IL-2 every 4 weeks led to a robust and sustained increase in the Treg population, along with enhanced expression of the IL-2 receptor, CD25, and the Treg transcription factor, FoxP3. This was accompanied by an improvement in Treg suppressive function, with no off-target effects on T effector lymphocytes, providing evidence of target engagement of low-dose IL-2 administration on Treg population. The range of changes from baseline in Treg percentage following IL-2 administration every four weeks in our study is comparable to that observed in other recently published trial using a similar 5-day low-dose IL-2 administration protocol [35]. Interestingly, increasing the frequency of IL-2 administration to every two weeks had a diminished effect on Treg expansion and the maintenance of FoxP3 expression, from day 92 to the end of the treatment phase on day 148. Repeated stimulation of Tregs without sufficient rest periods has been reported to result in exhausted and unstable Treg populations [36–38]. While these exhausted Tregs remain suppressive in vitro, they may lose their immunomodulatory functions in vivo [37–39], potentially accounting for the diminished impact on modifying inflammatory mediators, AD biomarkers and clinical decline in the IL-2 q2wks arm.

In the longitudinal analysis of plasma mediators, IL-2 q4wks administration suppressed inflammatory chemokines CCL2 and CCL11, as well as cytokine IL-15. CCL2 and CCL11 are two well-known myeloid activating analytes that robustly trigger the migration and infiltration of peripheral monocytes and central microglia toward injured areas and promote the release of reactive oxygen species. Additionally, IL-15 induces T cell activation and differentiation into effector cells [40–43]. The detrimental effects of these inflammatory mediators in exacerbating neuroinflammation and AD pathology have been well-documented, and various strategies targeting these mediators have been proposed as potential treatments for neurodegenerative disorders [40, 41, 44–48]. In addition to suppressing these inflammatory mediators, IL-2 q4wks treatment enhanced the anti-inflammatory cytokine IL-4, which may shift peripheral T cells toward an anti-inflammatory Th2 profile and promote CNS microglial activation toward an alternative, reparative phenotype [49, 50]. This shift could facilitate improved clearance of amyloid-beta and reduce neuroinflammation, potentially benefiting AD pathology and cognitive outcomes [51, 52]. IL2 q4wk administration also increased the plasma level of CCL13 mainly following IL2 q4wks treatment.

While the mechanism of CCL13 in modulating inflammatory responses in AD pathogenesis is not clear, growing evidence suggests that CCL13 and IL4 are closely linked to Th2 mediated immune responses and the activation of eosinophils [53, 54]. Consistent with pattern of changes in Treg percentage and Foxp3 MFI, increasing the frequency of IL-2 administration cycles to every two weeks reduced the efficacy of this treatment strategy in modifying systemic immune-related biomarkers. These findings further support the hypothesis that excessively frequent IL-2 administration might lead to the development of exhausted and unstable Treg populations with less effective modulation of plasma immune-related biomarkers. In an ongoing study, to better understand how different frequencies of low-dose IL-2 immunotherapy shape immune cell phenotypes and influence systemic and central proteomic landscapes, integrated single-cell transcriptomic and proteomic analyses will be applied to samples obtained from participants enrolled in this trial.

Regarding the impact of low-dose IL-2 treatment on established AD biomarkers and clinical scales, 21 weeks of IL-2 q4wks administration sustainabily increased number of Tregs, improved CSF soluble Aβ42 levels (a possible indicator of decreased amyloid β pathology) and showed promising trends on clinical endpoints. These outcomes are consistent with preclinical findings from our group and others, and with our previous clinical findings [14, 28, 29, 55, 56]. In our most recent preclinical study, peripheral Treg therapy downregulated CNS pro-inflammatory mediators, suppressed activated glial cells and reduced amyloid burden [14]. In the same context, low-dose IL-2-induced Treg expansion in other AD mouse models enhanced neuroprotection against AD pathology and restored cognitive function [28]. In an initial phase 1 feasibility study, IL-2 q4wk-induced Treg expansion effectively suppressed peripheral myeloid populations and decreased the level of systemic inflammatory cytokines and chemokines [29]. However, the impact of this treatment strategy on central inflammation in the clinical setting of AD remains unclear. In this regard, further studies in AD subjects are warranted to uncover the potential systemic and central mechanisms modulated by IL-2 immunotherapy and to explore their links to AD pathology.

## Conclusion

The IL-2 immunotherapeutic treatment regimens administered in this study were safe and well-tolerated. Administering 5-day low-dose IL-2 cycles every 4 weeks effectively and sustainably expanded Treg populations without off-target effects on effector CD4 or CD8 populations. IL2 q4wks administration also suppressed plasma inflammatory mediators, including CCL2, CCL11, and IL-15, while enhancing plasma levels of the anti-inflammatory cytokine IL-4. Although this trial was not primarily designed to assess the impact of low-dose IL-2 on AD biomarkers or cognitive outcomes, promising trends were found toward modifying AD progression with IL-2 administration every 4 weeks. Small sample size, short treatment duration and limited post-treatment follow-up period are limitations of this study. In this regard, a larger and longer clinical trial is warranted to further evaluate the efficacy of low-dose IL-2 as a potential therapeutic strategy for AD.

## Supplementary Information

Supplementary Material 1.

## Data Availability

The datasets supporting the conclusions of this article are included within the article and its additional supplementary file.

## Abbreviations

Tregs: Regulatory T cells
AD: Alzheimer’s disease
IL-2: Interleukin-2
IL-2 q4wks: Interleukin-2 every 4 weeks
IL-2 q2wks: Interleukin-2 every 2 weeks
CSF: Cerebrospinal fluid
CCL2: C–C motif chemokine ligand 2
CCL11: C–C motif chemokine ligand 11
IL-15: Interleukin 15
IL-4: Interleukin 4
Aβ42: Amyloid beta-peptide 1–42
NfL: Neurofilament light chain
ADAS-cog: Alzheimer’s Disease Assessment Scale Cognitive Subscale
CCL4: C–C motif chemokine ligand 4
CNS: Central nervous system
MMSE: Mini-Mental State Examination
p-tau181: Phosphorylated tau 181
GFAP: Glial fibrillary acidic protein
CDR-SB: Clinical Dementia Rating Scale Sum of Boxes
ADCS-CGIC: Alzheimer’s Disease Cooperative Study-Clinical Global Impression of Change
ANCOVA: Analysis of covariance
MFI: Mean fluorescence intensity
MMRM: Mixed mode for repeated measures
LS: Least Square
NKC: Natural killer cells
