# Supplementary material for: Low-dose interleukin-2 in patients with mild to moderate Alzheimer’s disease: a randomized clinical trial
Source: Alzheimers Res Ther. 2025 Jul 4;17:146. doi: 10.1186/s13195-025-01791-x (PMC12231701; doi:10.1186/s13195-025-01791-x)

**eFigure-1:** **Flow cytometry gating strategy**. Lymphocytes were first gated based on size and granularity using FSC and SSC parameters **(A)**, followed by gating on singlets **(B)** and live cells **(C)**. CD3 was used to distinguish T cells (CD3+) from non-T cells (CD3-) **(D)**. The CD3 negative population was further gated on CD56+ to define CD56NK **(E)**. The CD3 positive population was subsequently divided into CD8 T cells **(F)** and CD4 T cells **(G)**. The CD4 T population was then gated on CD25+ **(I)**, with gating controlled by a CD25 FMO **(H)**. CD4 Treg were defined by high expression of CD25 and intracellular FOXP3 **(K)**, with gating controlled by a FOXP3 FMO **(J)**.


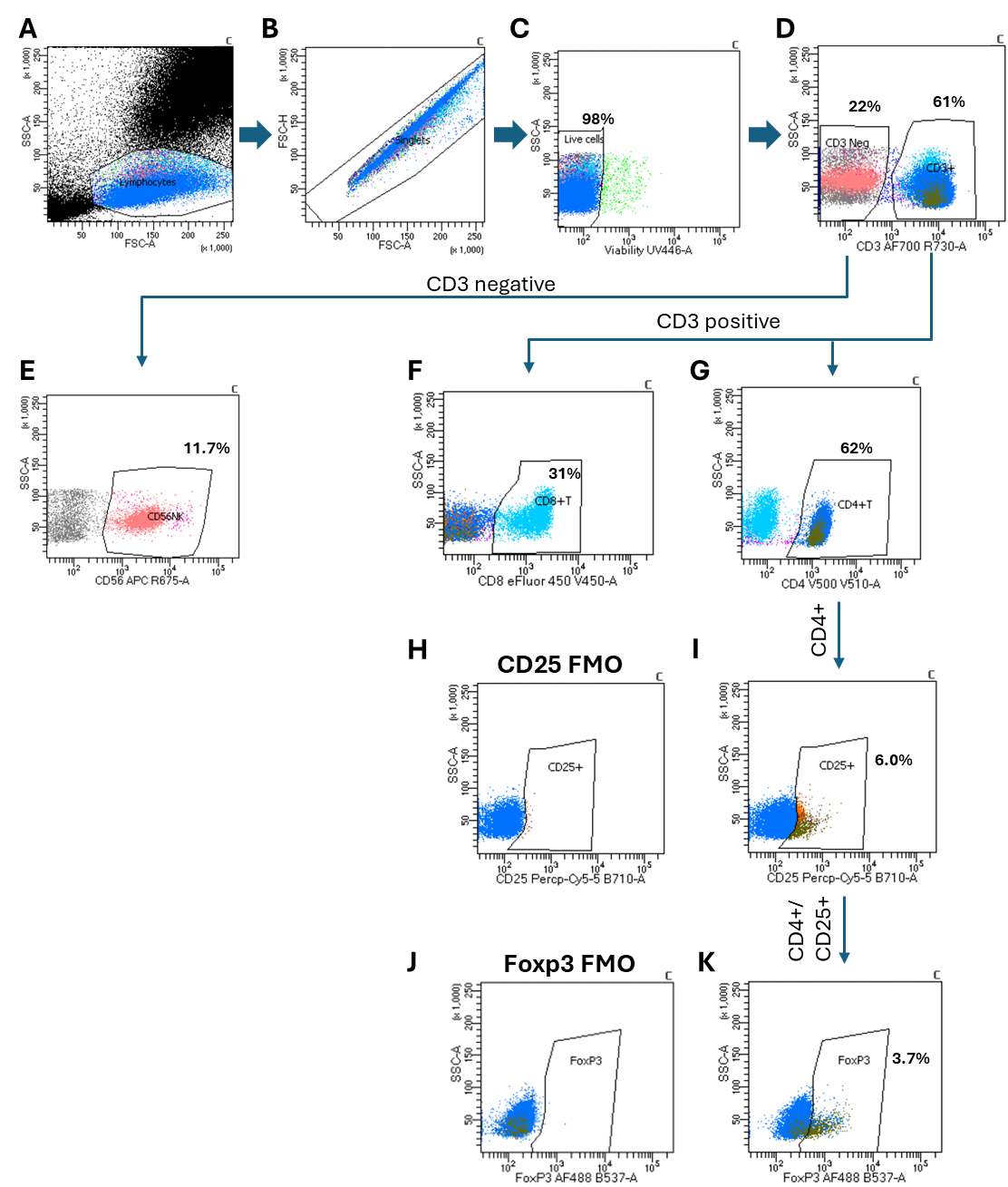


**eTable-1: Comparison of Baseline demographic characteristics of the clinical trial participants in each treatment arm compared to corresponding placebo group.**

| **Characteristics** | **IL-2 q 4wks (n=9)** | **Corresponding Placebo**  **(n=13)** | **p-Value q4wks vs. placebo** | **IL-2 q 2wks**  **(n=10)** | **Corresponding Placebo**  **(n=6)** | **p-Value** |
| --- | --- | --- | --- | --- | --- | --- |
| **Age-yr (MEAN)** | 68.7 | 68.8 | 0.975 | 75.9 | 68.5 | 0.068 |
| **Sex- no. (%)** |  |  |  |  |  |  |
| Female | 6 (66.6%) | 8 (61.54%) | 1.000 | 6 (60.0%) | 3 (50%) | 1.000 |
| Male | 3 (33.3%) | 5 (38.46%) |  | 4 (40.0%) | 3 (50%) |  |
| **Race/ Ethnicity-no. (%)** |  |  |  |  |  |  |
| White/non-Hispanic | 9 (100%) | 10 (76.92%) | 0.493 | 9 (90.0%) | 6 (100%) | 1.00 |
| White/ Hispanic | 0 (0%) | 2 (15.38%) |  | 1 (10.0%) | 0 (0.00%) |  |
| Black | 0 (0%) | 1 (7.69%) |  | 0 (0.0%) | 0 (0.00%) |  |
| **Education- no. (%)** |  |  |  |  |  |  |
| High school | 0 (0%) | 2 (15.38%) | 0.120 | 2 (20.0%) | 0 (0%) | 0.752 |
| College | 6 (66.6%) | 7 (53.58%) |  | 6 (60.0%) | 5 (83.33%) |  |
| Post-grad | 3 (33.3%) | 4 (30.77%) |  | 2 (20.0%) | 1 (16.67%) |  |
| **APOE ε4 status- no. (%)** |  |  |  |  |  |  |
| Noncarrier | 3 (33.3%) | 4 (30.77%) | 1.000 | 2 (20.0%) | 2 (33.33%) | 0.065 |
| Carrier-Heterozygotes | 4 (44.4%) | 5 (38.46%) |  | 6 (60.0%) | 0 (0.00%) |  |
| Carrier-Homozygotes | 2 (22.2%) | 4 (30.77%) |  | 2 (20.0%) | 4 (66.67%) |  |
| **Cognitive scales (MEAN±SD)** |  |  |  |  |  |  |
| MMSE score | 18.7 | 16.8 | 0.975 | 18.5 | 18.5 | 1 |
| CDR-SOB score | 4.7 | 4.6 | 0.939 | 4.3 | 5.3 | 0.445 |
| ADAS-Cog score | 31.2 | 35.3 | 0.552 | 30.7 | 37.3 | 0.209 |

**eTable-2: The means of safety laboratoy values at baselina and at the end of the treatment phase in clinical trial participants.**

**eTable-3:** Post-hoc analysis comparing Treg variables, CSF AD biomarkers, and cognitive functions was conducted in all participants who received IL-2 treatment (IL-2 q2wks and IL-2 q4wks combined) versus all placebo participants, as well as between each active treatment arm and its respective placebo arm.**
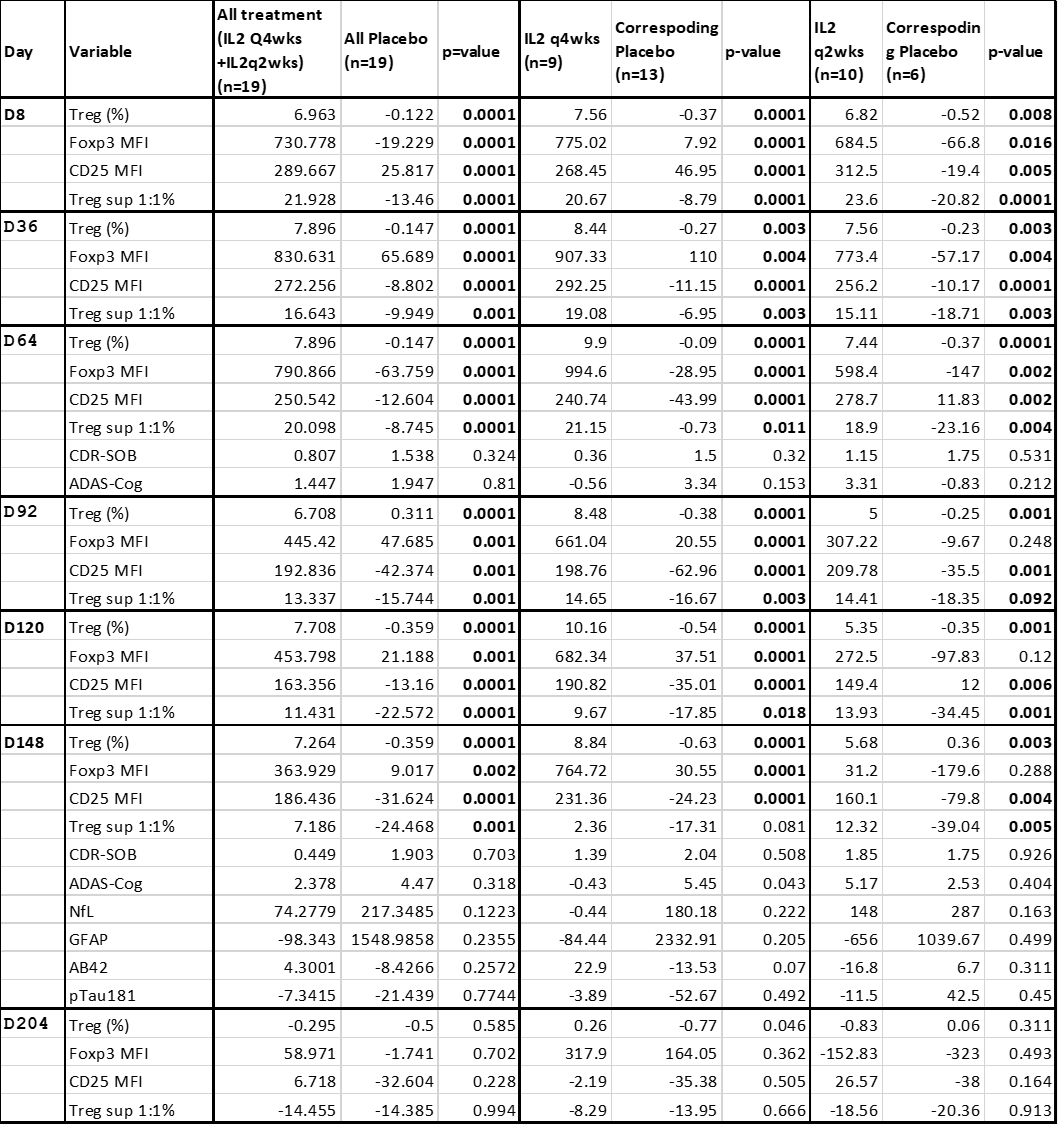
**

**eFigure 2:** **Effect of IL-2 Treatment on lymphocytes and Natural Killer Cells.** The percentage of CD4^+^CD25^low^ T responder cells (Tresp) **(A)**, CD8^+^ T cells **(B)**, and CD3^-^CD56^+^ natural killer cells (NKC) was assessed by flow cytometry at baseline (D1), on D8, D36, D64, D92, and D120 during the treatment phase, and at the end of the follow-up (F/U) phase at D204 across three study arms: IL-2 every 4 weeks (IL-2 q4wks), IL-2 every 2 weeks (IL-2 q2wks), and placebo. D = Day. Data represent mean changes from baseline± SE. Mean changes from baseline across the three arms were compared using ANCOVA. P-values are represented as *p < 0.05.


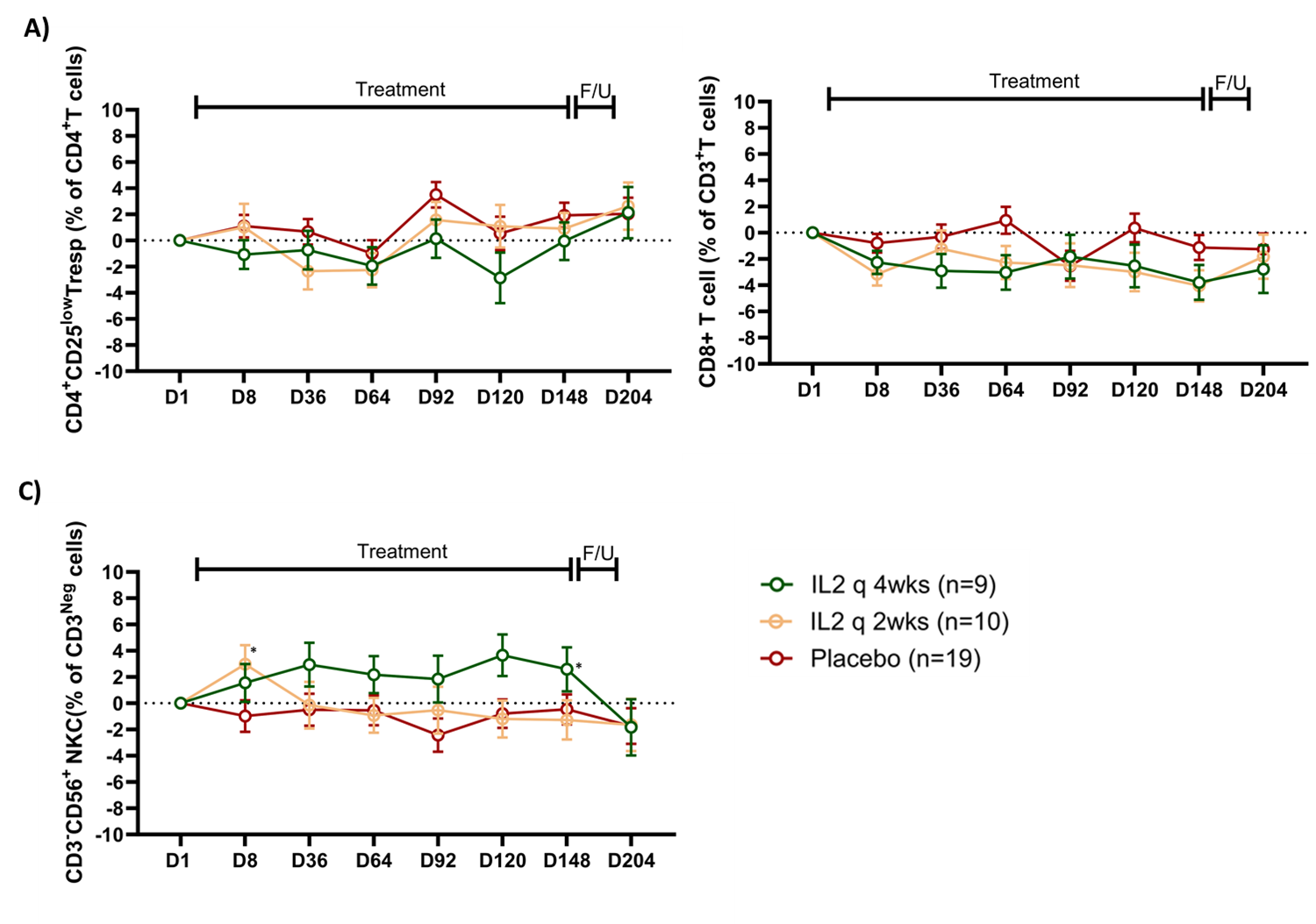


**efigure-3: Effect of low-dose IL-2 treatment on plasma inflammatory biomarkers.** No statistically significant longitudinal changes were observed in 31 other measured plasma immune markers using Olink protein analysis at baseline (D1), throughout the treatment phase (D8, D64, D92, D120, and D148), and at the end of the follow-up (F/U) phase on D204 across three study arms: IL-2 every 4 weeks (IL-2 q4wks), IL-2 every 2 weeks (IL-2 q2wks), and placebo. D = Day. Data represent mean changes from baseline ± SE. Comparison across the three arms were compared using ANCOVA.


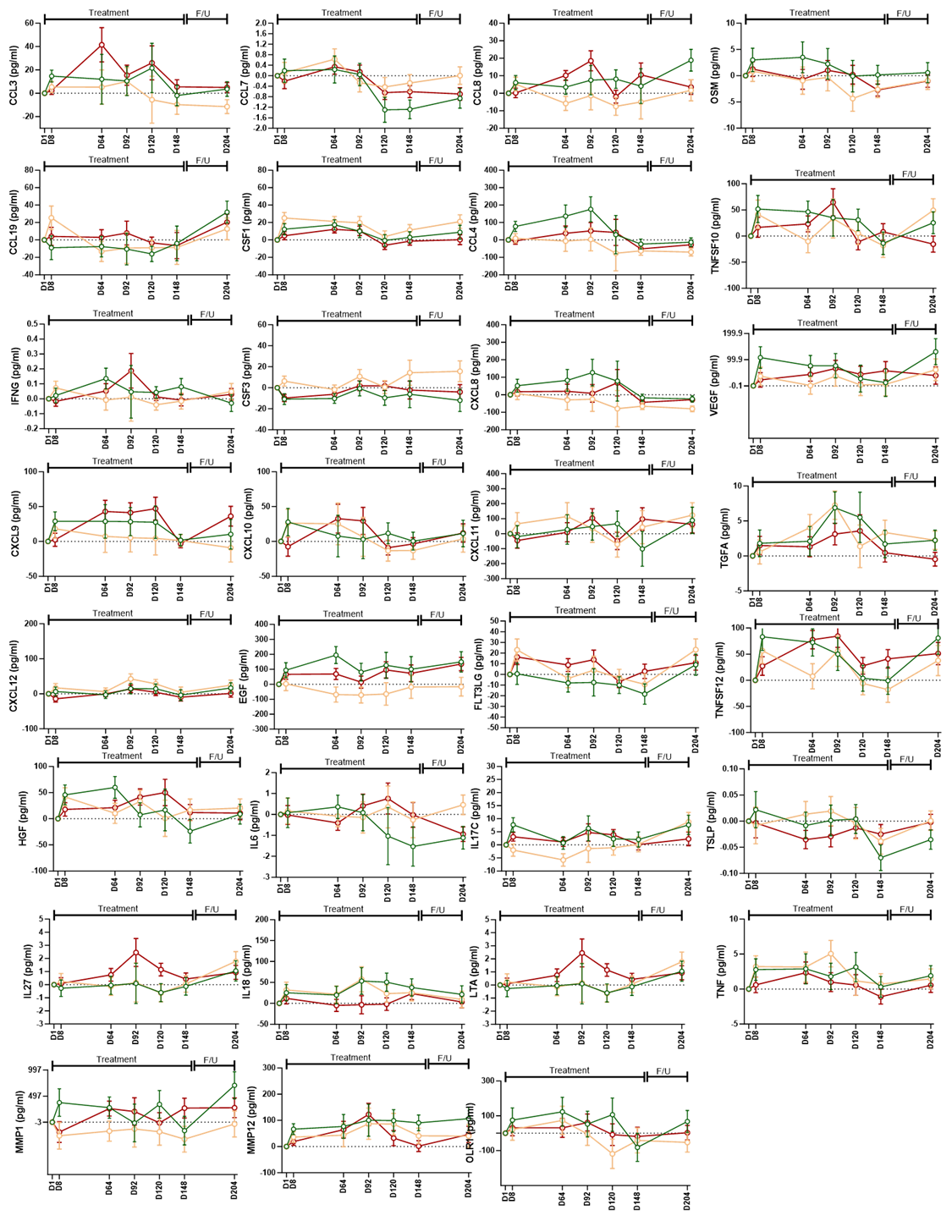


**efigure 4: Effect of Low-Dose IL-2 Treatment on Clinical Scales during the treatment and also in extended post-treatment follow up phase.** Cognitive tests, including the Alzheimer's Disease Assessment Scale–Cognitive Subscale (ADAS-Cog) (A), Clinical Dementia Rating Scale Sum of Boxes (CDR-SB) (B), were conducted at screening (SC), day 64 (D64), day 148 (D148) and 8 weeks after completion of the therapy on Day 204 (D204). Data represents mean changes from baseline ± SE.


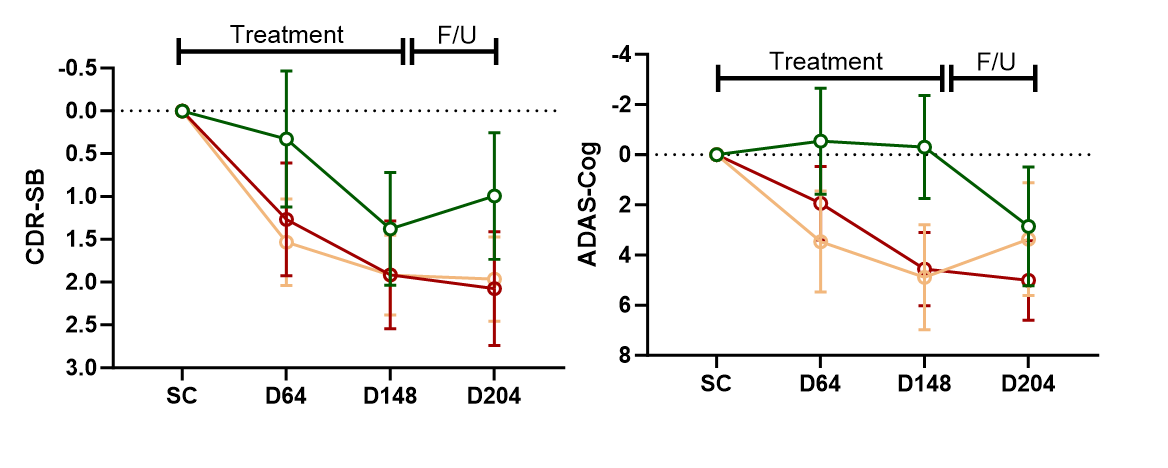

Supplement: Supplementary file 1 — Supplementary Material 1. [file 13195_2025_1791_MOESM1_ESM.docx]
